# Look for the Union Label

**Published:** 1907-12

**Authors:** 


### "Look for the Union Label."

There is a strenuous effort to trade-unionize the American medical profession, even to the extent of requiring the "union label" on all medicinal products, in the form of the indorsement of the Council of Pharmacy and Chemistry of the American Medical Association. Read the following in the March number of the *California State Journal of Medicine:*

"Any remedial preparation that you do not find in the list of 'new and non-official remedies,' as issued by the Council, is one to look upon with suspicion; it *may* be a good and legitimate product, but the chances are that it is not, or that the proprietors have uttered exaggerated statements as to its value."

Are we to assume from this that the Council or its misadvised friends propose to institute a boycott against all medicinal products, whose manufacturers refuse to be fitted to the Procrustean bed which has been especially built for them? It looks so—in spite of the fact that when the Council was established it was distinctly stated that the absence of any product from its "extra-pharmacopeia" was not to be regarded as prejudicial to such product. It looks now as if the great Association proposes to raise the cry of "scab" against every manufacturer of sufficient independence of character to refuse to conform to the exactions of the Council, some of which are inane and unjust.

While studying over this proposed boycott, it is worth your while to bear in mind the following facts: That of the 250 odd preparations thus far "passed" by the Council, less than 60 are American; the balance are foreign products, mostly coal-tar synthetics. There are dozens and dozens of these of which the average American physician has never even heard, and whose therapeutic uses are but imperfectly known. Furthermore, please note that of the 59 American products admitted, *one house* has 23. The following American firms are not represented—not even by a single product: H. K. Mulford & Co., John Wyeth & Co., McKesson & Robbins, William R. Warner & Co., Eli Lilly & Co., William S. Merrill Chemical Company, Lloyd Brothers, The Abbott Alkaloidal Company, and also practically all of the proprietary manufacturers, good, bad, or indifferent—in fact, we can now think of but one of the last which has been let in, and that one with a product which is a spy employe pirated imitation of antiphlogistine.

It is also worth while to remember that of the fifteen members of the Council *not one is a practicing physician.* Practically all are pharmacists, pharmaceutical teachers or chemists, not one of whom is fitted, by practical experience, to pass upon the therapeutic efficiency of a single preparation; and yet they can "blackball" on the charge of "exaggeration"—as well as upon a number of other points having no bearing upon the merit of the preparation whatever. Yet the findings of the Council are to be made the basis of a systematized attack upon all manufacturers who decline to apply for admission, for any reason whatsoever, to the "extrapharmacopeia"—is that what we are to understand? Let us have a frank statement of your purpose, gentlemen. It will clear the atmosphere.—*Clinical Medicine.*

---

Guaiacol Cinnamate.—In the intestinal tract, guaiacol cinnamate becomes decomposed and liberates the guaiacol. The powder is said to be without smell or taste, and has been employed in obstinate diarrheas and at the beginning of tuberculosis. It is given in doses of 1 gramme daily, in four doses, to children at the breast, or 1 gramme 50 centigrammes to older children. Adults can take 1 gramme four times a day.—*Journal de médecine de Paris.*

